# Cardiac magnetic resonance T1 mapping for amyloid cardiomyopathy in patients with heart failure

**DOI:** 10.1093/ehjimp/qyag131

**Published:** 2026-07-23

**Authors:** Lara Unger, Romy Gessner, Tina Stegmann, Andreas Hagendorff, Solveig Tiepolt, Osama Sabri, Sebastian Ebel, Timm Denecke, Ulrich Laufs, Daniel Lavall

**Affiliations:** Department of Cardiology, Leipzig University Hospital, Liebigstrasse 20, Leipzig 04103, Germany; Central German Heart Alliance, Germany; Department of Cardiology, Leipzig University Hospital, Liebigstrasse 20, Leipzig 04103, Germany; Central German Heart Alliance, Germany; Department of Cardiology, Leipzig University Hospital, Liebigstrasse 20, Leipzig 04103, Germany; Central German Heart Alliance, Germany; Department of Cardiology, Leipzig University Hospital, Liebigstrasse 20, Leipzig 04103, Germany; Central German Heart Alliance, Germany; Department of Nuclear Medicine, Leipzig University Hospital, Leipzig, Germany; Department of Nuclear Medicine, Leipzig University Hospital, Leipzig, Germany; Department of Diagnostic and Interventional Radiology, Leipzig University Hospital, Leipzig, Germany; Department of Diagnostic and Interventional Radiology, Leipzig University Hospital, Leipzig, Germany; Department of Cardiology, Leipzig University Hospital, Liebigstrasse 20, Leipzig 04103, Germany; Central German Heart Alliance, Germany; Department of Cardiology, Leipzig University Hospital, Liebigstrasse 20, Leipzig 04103, Germany; Central German Heart Alliance, Germany

**Keywords:** ATTR, transthyretin amyloid cardiomyopathy, left ventricular hypertrophy, cardiac magnetic resonance imaging, heart failure

## Abstract

**Aims:**

This study investigates the diagnostic accuracy of cardiac magnetic resonance (CMR) T1 mapping for the evaluation of cardiac amyloidosis (CA) in elderly patients with heart failure (HF).

**Methods and results:**

We prospectively enrolled consecutive patients aged ≥60 years with symptomatic HF, left ventricular ejection fraction (LVEF) ≥ 40%, increased left ventricular (LV) wall thickness (≥12 mm), and elevated cardiac biomarkers. All patients underwent standardized CMR, bone scintigraphy, and screening for monoclonal proteins. Primary endpoint was the diagnosis of CA with T1 mapping. The reference standard was a confirmed CA diagnosis by bone scintigraphy. One hundred and twelve patients (median age 81 years, 45% female) were included. CA was diagnosed in 38 patients (34%). Patients with CA showed advanced LV remodelling, indicated by higher LV mass index, lower LVEF, higher native T1 values, and higher extracellular volume fraction (ECV). Diagnostic accuracy on ROC curve analysis was 0.836 for native T1 and 0.904 for ECV. Native T1 < 1303 ms excluded CA. In patients with native T1 ≥ 1303 ms, there was no CA as cause of HF observed if ECV < 30%, while 85% of patients with an ECV 40–49% and 100% with ECV ≥ 50% had CA (*P* < 0.0001). By this approach, contrast application could have been avoided in 33%. A definite confirmation or exclusion of CA by CMR was established in 50%.

**Conclusion:**

In elderly patients with symptomatic HF, LVEF ≥ 40%, LV hypertrophy, and elevated cardiac biomarkers, a CMR approach with native T1 and ECV measurements is useful to identify underlying CA.

**Trial registration:**

NCT04862273 at clinicaltrials.gov.

## Graphical Abstract

**Graphical Abstract:**
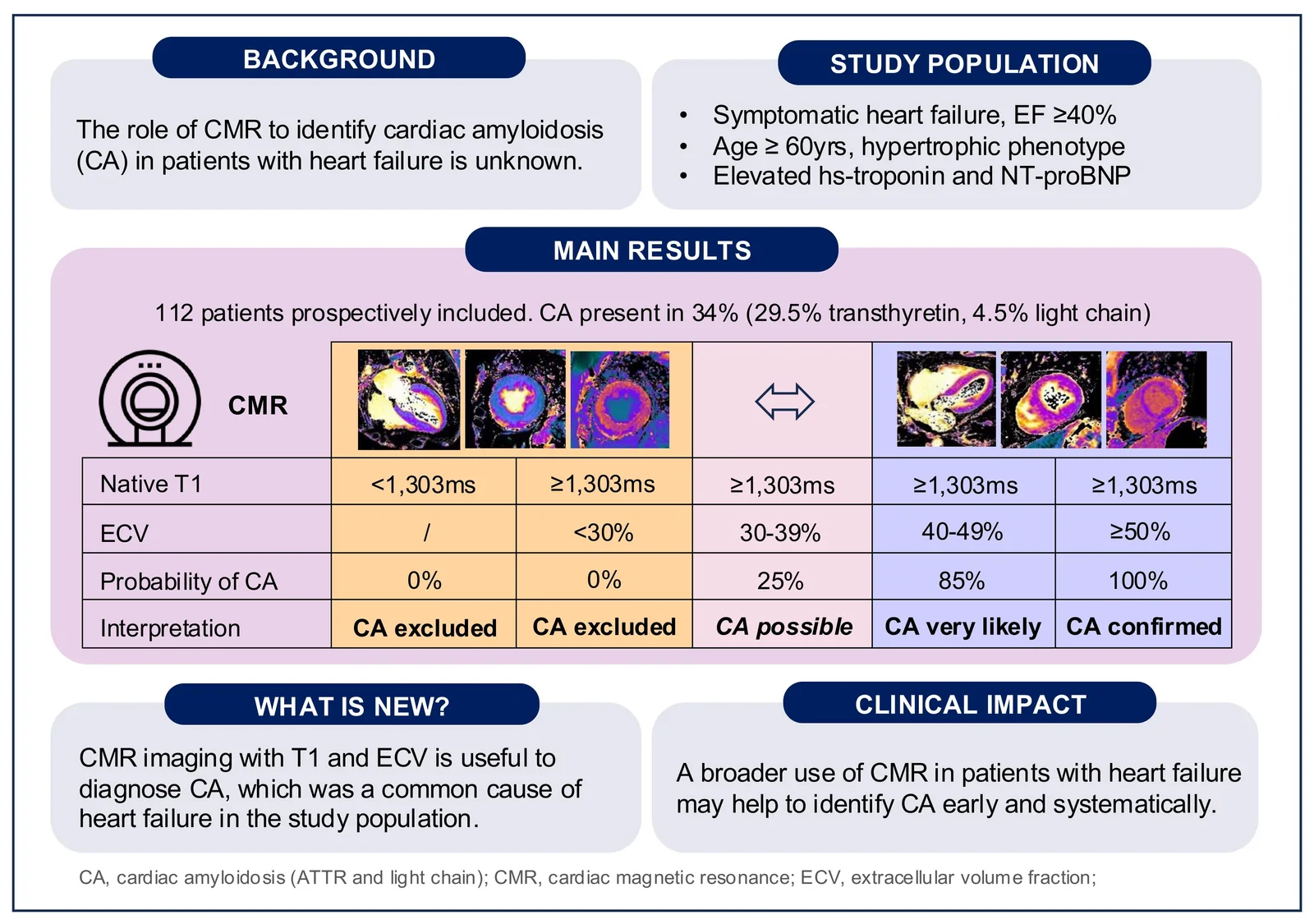

## Introduction

Cardiac amyloidosis (CA) is a myocardial disease with interstitial deposition of amyloid fibrils leading to an increased left ventricular (LV) mass. Heart failure (HF) with poor prognosis is often the result of the typical restrictive pathophysiology in patients with CA.^1,2^ Most common types of CA are light chain amyloidosis (AL) and transthyretin amyloidosis (ATTR).^3^ The estimated prevalence of transthyretin amyloid cardiomyopathy (ATTR-CM) is about 14% in elderly patients with HF with preserved ejection fraction and increased LV wall thickness.^4,5^ However, CA is supposed to be an underdiagnosed entity due to the lack of specific symptoms and signs. Timely diagnosis of CA is of particular importance as current therapies aim to stop progression of the disease.^6,7^

Bone scintigraphy and screening for monoclonal proteins are the non-invasive standard to diagnose CA.^7–10^ However, scintigraphy is limited by exposure of radiation, inability of myocardial tissue characterization for differential diagnoses, and the need of nuclear imaging expertise. Thus, the diagnosis of amyloid cardiomyopathy requires additional cardiac imaging such as echocardiography or cardiac magnetic resonance (CMR). The strength of CMR is to characterize and to quantify myocardial remodelling and function. Native T1 mapping is a sensitive method to detect diffuse myocardial interstitial alterations and to differentiate causes of LV hypertrophy.^11–14^ The administration of contrast enhances diagnostic power.^15^ Previous studies demonstrated higher myocardial T1 values in CA compared with other causes of LV hypertrophy in highly selected patients with suspected amyloidosis in specialized centres.^15–18^ We recently reported elevated native T1 values in patients with CA compared with hypertrophic cardiomyopathy and hypertensive heart disease in a retrospective analysis.^19^ The value of CMR for a systematic evaluation of CA among patients with HF is unknown.

The aim of this study was to evaluate the diagnostic accuracy of T1 parametric imaging for the diagnosis of CA (both ATTR and AL) in elderly patients with symptomatic HF and additional risk factors for CA.

## Methods

### Patient study population

**Figure 1:**
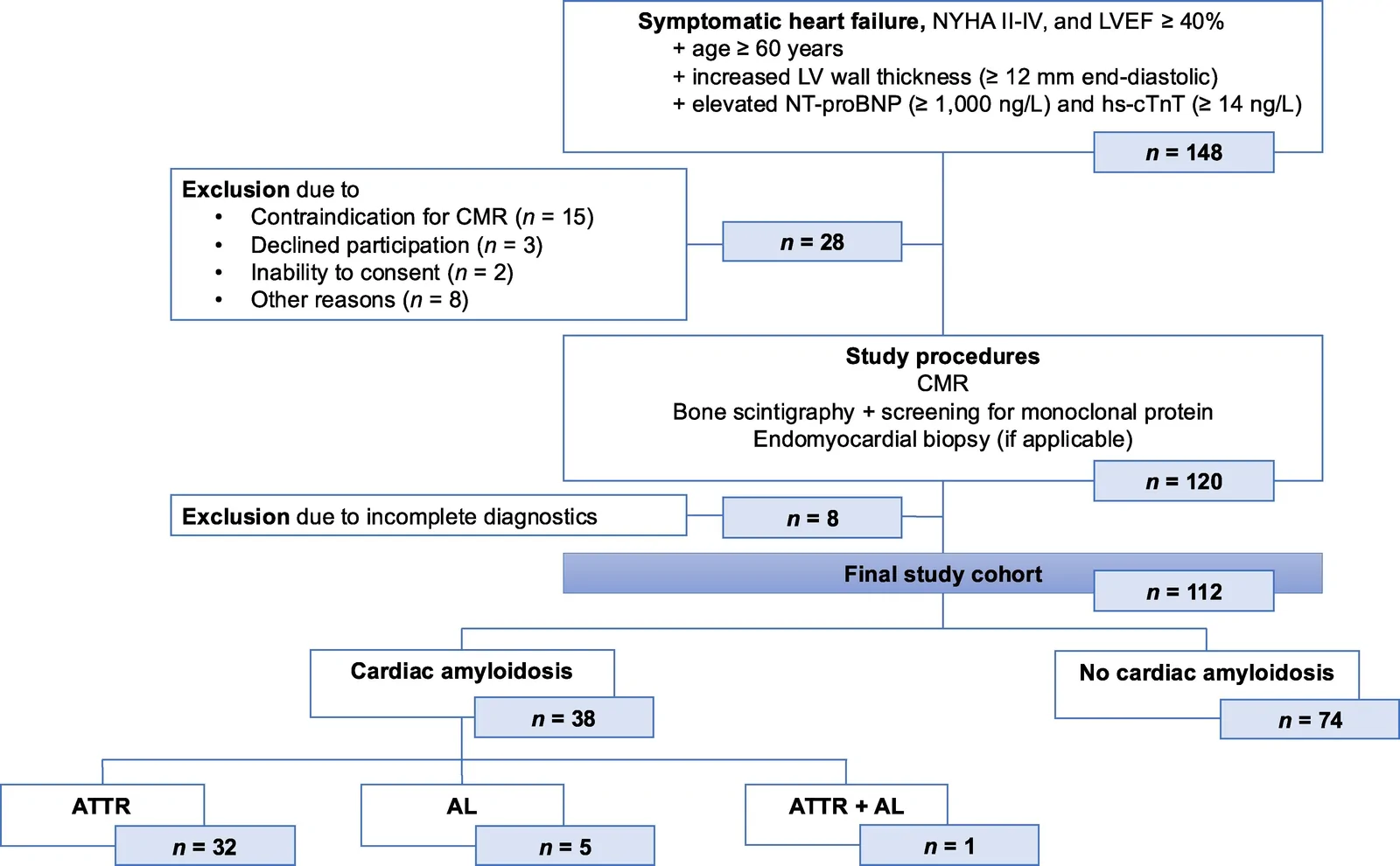
Study flow chart. Included patients underwent quantitative CMR, bone scintigraphy with SPECT/CT, and screening for monoclonal protein. AL, light chain amyloidosis; ATTR, transthyretin amyloidosis; CMR, cardiac magnetic resonance; hs-cTnT, high-sensitivity cardiac troponin T; LV, left ventricular; LVEF, left ventricular ejection fraction; NT-proBNP, N-terminal pro-B-type natriuretic peptide; NYHA, New York Heart Association.

We prospectively enrolled consecutive patients at the Leipzig University Hospital who presented with symptomatic HF (≥functional New York Heart Association class II), left ventricular ejection fraction (LVEF) ≥ 40%, LV end-diastolic wall thickness ≥ 12 mm on echocardiography, age of 60 years or older, and increased cardiac biomarkers [N-terminal pro-B-type natriuretic peptide (NT-proBNP) ≥ 1000 ng/L and high-sensitivity cardiac troponin T (hs-cTnT, Elecsys assay, Roche Diagnostics) ≥ 14 ng/L] (*Figure 1*). Inclusion criteria were based on previous studies focusing on patients at high risk for CA. Patients with contraindications to CMR, acute myocarditis, or acute myocardial infarction within 1 month prior to enrolment were excluded because of increased T1 values in these situations.^11^ Patients with severe aortic stenosis were eligible if Remodelling, Age, Injury, System, and Electrical (RAISE) score were ≥ 2 points (see Supplementary data online, *Table S1*).^20^ The sample size calculation is provided in the Supplementary data.

This study complies with the *Declaration of Helsinki* and was approved by the local ethics committee of Leipzig University (number 149/21-ek). The study is registered at Clinicaltrials.gov (study ID: NCT04862273).

### Study procedures and outcomes

All patients underwent technetium-99m-labelled 3,3-diphosphono-1,2-propanodicarboxylic acid ([^99m^Tc]DPD) bone scintigraphy with SPECT/CT (reference method), blood analysis for monoclonal proteins, and CMR (*Figure 1*). Cardiac ATTR was defined by a Perugini score of 2 or 3 in bone scintigraphy and a negative screening for monoclonal proteins. If imaging results were inconclusive (e.g. Perugini score 1) or monoclonal protein was present, endomyocardial biopsies from the LV were obtained.^7–10, 21^ AL amyloidosis was confirmed by tissue biopsy. In patients without CA, the final diagnosis was established by means of clinical and imaging data as well as medical history (*post hoc*).

The primary outcome was the diagnostic accuracy of T1 imaging for the diagnosis of CA by CMR. For the diagnosis of CA, we analysed T1 mapping in short axis (SAX), T1 mapping in four-chamber (4Ch) orientation, and the extracellular volume fraction (ECV).

### CMR protocol and analysis

CMR was performed using a Philips Achieva 3.0 Tesla scanner (Philips Healthcare) with a structured study protocol including cine sequences in short and long axes, native and post-contrast T1 imaging, and late gadolinium enhancement (LGE), if applicable. Native and contrast-enhanced T1 sequences were obtained in three SAX (basal, mid, and apical) as well as in one 4-chamber slice. LGE was assessed at least 10 min after intravenous contrast application (0.15 mmol/kg body weight gadobutrol, Bayer) in 95 patients (severe chronic kidney disease was considered as contraindication). Details of CMR are provided in the Supplementary data.

CMR data analyses were performed offline with IntelliSpace Portal software (Philips Healthcare) according to current recommendations.^13^ End-diastolic and end-systolic LV diameters and inter-ventricular septal and posterior wall thickness were measured in a mid-ventricular SAX. LV volumes, mass, stroke volume, and LVEF were determined in SAX stack covering the entire LV. Papillary muscles were automatically subtracted from the blood cavity. LV mass-to-volume index (MVI) was calculated as LV mass/end-diastolic volume. Left atrial (LA) volumes were calculated from the end-systolic LA areas in two-chamber and in 4Ch orientation. LGE was measured qualitatively (any vs. no LGE presence) and quantitatively in a SAX LV stack based on endocardial and epicardial border contours and adjusted manually if necessary. Pathologic LGE was assumed if the LGE signal was more than three-fold greater than the standard deviation of a reference myocardial segment free of LGE. Results are depicted as percentage of LGE in the LV myocardium.

Since T1 measurements are not standardized, we obtained three native T1 values, which were used in previous studies: (i) global T1 value from three SAX slices (basal, mid, and apical), each containing six segments; (ii) septal T1 value from three SAX slices (basal, mid, and apical); and (iii) T1 in 4Ch slice in the septal region. ECV was calculated from native and contrast-enhanced three SAX of the LV myocardium, with the measured haematocrit imputed into the software. Motion artefacts between native and post-contrast T1 measurements were corrected manually if necessary.

Interobserver/intraobserver variabilities [intraclass correlation coefficient with 95% confidence interval (CI)] for CMR measurements were 0.965 (0.844–0.991)/0.987 (0.945–0.997) for native T1 and 0.989 (0.952–0.997)/0.968 (0.877–0.992) for ECV.

### Performance of diagnostic test

To evaluate the diagnostic performance of CMR parameters for the diagnosis of CA (outcome variable), receiver operating characteristic (ROC) analyses were conducted. Area under the curve (AUC) values with 95% CI were calculated for each individual predictor. To determine the optimal threshold for each continuous predictor, the Youden’s index was applied and sensitivity, specificity as well as positive predictive values (PPV) and negative predictive values (NPV) reported. Additionally, PPV and NPV were calculated for an external pre-test probability for CA of 13%^4^ according to the formulae PPV = (Se × *P*) / ((Se × *P*) + (1 − Sp) × (1 − *P*)) and NPV = (Sp × (1 − *P*)) / ((Sp × (1 − *P*) + ((1 − Se) × *P*)), where Se is sensitivity, Sp is specificity, and *P* is pre-test probability.

We performed multivariable models for combining predictors using logistic regression analysis to create an optimal diagnostic algorithm. For the combined models, predicted probabilities were obtained and used to construct ROC curves. Bootstrap-corrected AUC values with 95% CI (*2000 stratified bootstrap replicates*) for the final algorithm are reported.

Sensitivity analyses using multiple imputation with both best- and worst-case scenarios were performed for ECV. Diagnostic improvement by the combined model compared with a single modality (T1 4Ch and ECV) was assessed by using continuous net reclassification improvement, calculated separately for events and non-events.

### Multivariate logistic regression analysis

To identify independent CMR predictors of CA, we performed multivariate logistic regression models for ECV and T1 values adjusting for clinical confounders, i.e. age, sex, eGFR, and hs-cTnT. Results are reported as odds ratios (OR) with corresponding 95% CI.

### Sensitivity analyses and robustness of the model

To assess the robustness and generalizability of the multivariable diagnostic model, cross-validation analyses were performed including leave-one-out cross-validation and *k*-fold cross-validation (with *k* = 10). In each fold, the model was trained on 90% of the data and tested on the remaining 10%, and cross-validated AUC values were computed as an estimate of out-of-sample performance.

The Fagan nomogram approach was used to translate and visualize diagnostic accuracy into clinically interpretable post-test probabilities. Based on sensitivity and specificity derived from the optimal ROC cut-off (Youden’s index), likelihood ratios (LR^+^ and LR^−^) were calculated. These likelihood ratios were combined with the pre-test probability (based on sample prevalence) to estimate post-test probabilities for positive and negative test results.

### Statistical analyses

Continuous variables are expressed as mean ± standard deviation if normally distributed or as median and interquartile range. Differences between the two groups were analysed by unpaired Student’s *t*-test, Mann–Whitney *U*-test, or *χ*^2^ test, as appropriate, and ANOVA for multiple comparisons. Categories of ECV values were compared with Fisher’s exact test. AUC values between CMR parameters were compared with DeLong test. Differences with a *P*-value of <0.05 were defined as statistically significant. Statistical analyses were performed using Jamovi 2.4.8.0, GraphPad Prism 10, SPSS 29.0.0.0, and R (version 2026.01.0+392).

## Results

### Baseline characteristics

Between April 2021 and October 2023, 148 patients were screened, and 120 were enrolled in the study. Among those, eight participants were excluded due to incomplete diagnostics (refused biopsy in two patients, missing CMR, or scintigraphy data in six patients). Thus, the remaining 112 patients were included in the final analysis set.

The mean age was 81 years and 45% were female. CA was the final diagnosis by the reference method in 38 patients (34%). Among those, 32 (84%) patients suffered from cardiac ATTR, 5 patients (13%) had AL amyloidosis, and 1 patient had a combination of both ATTR and AL amyloidosis (*Figure 1*). Hypertensive heart disease (*n* = 65), severe aortic stenosis (*n* = 5), and hypertrophic cardiomyopathy (*n* = 4) were the predominant causes for LV hypertrophy without CA.

Patients with CA were predominantly males and had higher serum concentrations of hs-cTnT and NT-proBNP levels, higher LV wall thickness on echocardiography, and a higher prevalence of carpal tunnel syndrome and spinal stenosis (*Table 1*).

**Table 1:** Patient characteristics

	All patients (*n* = 112)	No amyloidosis (*n* = 74)	Amyloidosis (*n* = 38)	*P*-value amyloidosis vs. no amyloidosis
Age (years)	81.0 (74.0–85.0)	80.0 (71.3–84.8)	82.0 (77.0–84.8)	0.122
Female gender, *n* (%)	50 (45)	40 (54)	10 (26)	**0**.**005**
NYHA class				0.76
II, *n* (%)	42 (38)	26 (35)	16 (42)	
III, *n* (%)	63 (56)	43 (58)	20 (53)	
IV, *n* (%)	7 (6)	5 (7)	2 (5)	
Vital parameters				
Systolic blood pressure (mmHg)	142 ± 26	143 ± 26	140 ± 26	0.53
Diastolic blood pressure (mmHg)	78 ± 17	77 ± 17	79 ± 16	0.52
Heart rate (b.p.m.)	75 (65–85)	75 (64–89)	75 (67–82)	0.614
Body mass index (kg/m^2^)	25.4 (23.7–29.7)	26.3 (23.9–30.1)	24.3 (23.4–28.0)	0.155
Biomarkers				
NT-proBNP (ng/L)	3339 (2357–6508)	3179 (2007–5132)	4931 (2646–7027)	**0**.**037**
hs-cTnT (ng/L)	46.8 (31.4–80.9)	42.8 (28.8–62.8)	71.2 (46.2–102.0)	**<0.001**
eGFR (mL/min/1.73 m^2^)	48.7 ± 22.4	47.7 ± 22.5	50.7 ± 22.4	0.51
Electrocardiogram				
Sinus rhythm, *n* (%)	50 (45)	34 (46)	16 (42)	0.699
Atrial fib./flutter, *n* (%)	62 (55)	40 (54)	22 (58)	0.699
AV block, *n* (%)	18 (16)	11 (15)	7 (18)	0.553
BBB, *n* (%)	33 (29)	19 (26)	14 (37)	0.22
Low voltage, *n* (%)	15 (13)	3 (4)	12 (32)	**<0.001**
Echocardiography				
LV wall thickness (mm)	14.0 (13.0–15.0)	13.0 (12.3–14.8)	15.0 (14.0–17.0)	**<0.001**
Pericardial effusion, *n* (%)	20 (18)	11 (15)	9 (24)	0.249
Comorbidities				
Hypertension, *n* (%)	100 (89)	68 (92)	32 (84)	0.213
Diabetes mellitus, *n* (%)	49 (44)	38 (51)	11 (29)	**0**.**024**
Atrial fib./flutter, *n* (%)	80 (71)	53 (72)	27 (71)	0.95
CAD, *n* (%)	53 (47)	40 (54)	13 (34)	**0**.**046**
TIA/stroke, *n* (%)	17 (15)	9 (12)	8 (21)	0.214
PAD, *n* (%)	12 (11)	5 (7)	7 (18)	**0**.**004**
Extra-cardiac features of amyloidosis				
Orthostasis, *n* (%)	41 (37)	26 (35)	15 (39)	0.9
Syncope, *n* (%)	27 (24)	18 (24)	9 (24)	0.816
CTS, *n* (%)	26 (23)	11 (15)	15 (39)	**0**.**009**
Spinal stenosis, *n* (%)	27 (24)	12 (16)	15 (39)	**0**.**015**
Degen. joint disease, *n* (%)	28 (25)	15 (20)	13 (34)	0.106
NT tendon rupture, *n* (%)	3 (3)	1 (1)	2 (5)	0.273
Non-diabetic PNP, *n* (%)	10 (9)	4 (5)	6 (16)	0.095
Medication				
Anticoagulants, *n* (%)	79 (71)	53 (72)	26 (68)	0.882
Antiplatelets, *n* (%)	23 (21)	16 (22)	7 (18)	0.741
RAAS inhibitors, *n* (%)	95 (85)	63 (85)	32 (84)	0.848
Beta-blockers, *n* (%)	79 (71)	52 (70)	27 (71)	0.767
MRA, *n* (%)	23 (21)	17 (23)	6 (16)	0.408
SGLT-2 inhibitors, *n* (%)	41 (37)	31 (42)	10 (26)	0.126
Loop diuretics, *n* (%)	82 (73)	54 (73)	28 (74)	0.76

Patients in NYHA functional class I have not been included per protocol. Bold values indicate significant differences.

ATTR, transthyretin amyloidosis; AV block, atrioventricular block; BBB, bundle branch block; b.p.m., beats per minute; CAD, coronary artery disease; CTS, carpal tunnel syndrome; ECG, electrocardiogram; eGFR, estimated glomerular filtration rate; hs-cTnT, high-sensitivity cardiac troponin T; LV, left ventricular; LVEF, left ventricular ejection fraction; MRA, mineralocorticoid receptor antagonist; NT, non-traumatic; NT-proBNP, N-terminal pro-B-type natriuretic peptide; NYHA, New York Heart Association; PAD, peripheral arterial disease; PNP, polyneuropathy; RAAS, renin-angiotensin-aldosterone system; SGLT-2, sodium-glucose cotransporter-2; TIA, transient ischaemic attack.

**Figure 2:**
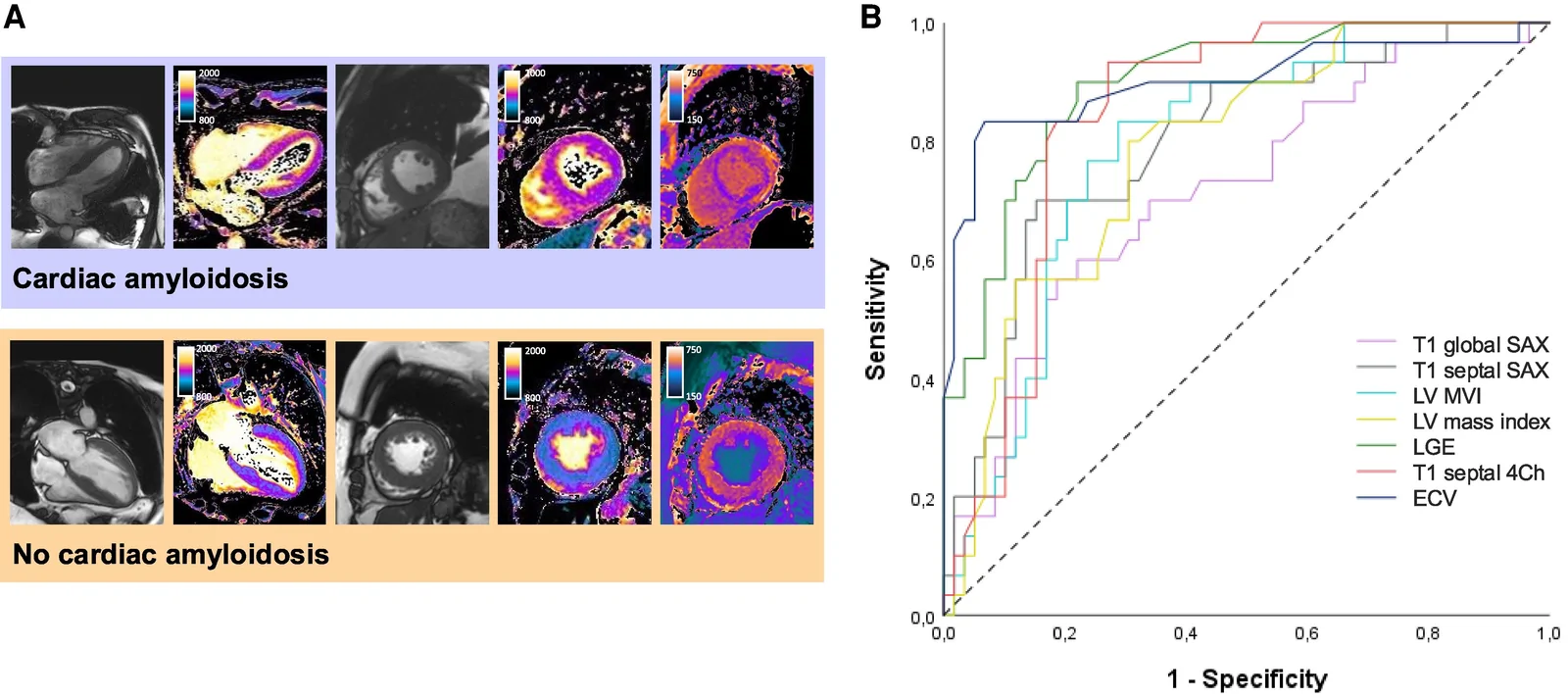
CMR parameters and their diagnostic accuracy for cardiac amyloidosis. (*A*) Representative examples of cine, native and contrast-enhanced T1 mapping images of patients with (first row, purple) and without (second row, orange) cardiac amyloidosis. (*B*) ROC curves of T1 global and septal SAX, T1 septal 4Ch, ECV, LGE, LV mass index, and LVMVI.

The CMR imaging results are presented in *Table 2*. CA was associated with significantly higher values of native T1, ECV, LGE, LV mass index, and LVMVI, indicating ATTR-CM. Representative CMR images for CA and non-CA are presented in *Figure 2A*.

**Table 2:** Cardiac magnetic resonance parameters

	No cardiac amyloidosis (*n* = 74, 66%)	Cardiac amyloidosis (*n* = 38, 34%)	*P*-value
IVSD (mm)	12.0 (11.0–14.0)	15.5 (13.3–20.0)	**<0.001**
LVPWD (mm)	8.0 (7.0–9.0)	9.0 (8.0–10.0)	0.07
LV wall mass index (g/m^2^)	52.5 (42.3–65.5)	74.5 (62.0–94.0)	**<0.001**
LVEDD (mm)	47.0 (43.0–50.0)	46.0 (42.3–49.8)	0.498
LVESD (mm)	29.0 (25.0–36.8)	29.5 (24.3–33.8)	0.82
LVEDV index (mL/m^2^)	47.5 (38.0–60.0)	47.5 (40.0–62.0)	0.448
LVESV index (mL/m^2^)	16.0 (12.0–27)	20.0 (15.0–28.0)	0.062
LVSV index (mL/m^2^)	30.0 (25.0–34.0)	26.5 (23.0–32.0)	0.276
LVMVI (g/mL)	1.12 (0.92–1.30)	1.56 (1.26–1.73)	**<0.001**
LVEF (%)	66.0 (53.0–73.0)	58.0 (50.0–64.0)	**0.007**
LAEDV index (mL/m^2^)	39.0 (25.5–50.5)	46.0 (24.0–52.0)	0.132
LAESV index (mL/m^2^)	48.0 (34.0–60.5)	52.5 (42.0–63.0)	0.285
T1 global (ms)	1262 ± 5	1322 ± 77	**<0.001**
T1 septal, short axis (ms)	1243 ± 83	1328 ± 81	**<0.001**
T1 septal, 4Ch (ms)	1330 ± 82	1429 ± 62	**<0.001**
ECV (%)	32.0 (28.3–33.8)	45.0 (39.0–51.5)	**<0.001**
LGE qualitative, *n* (%)	44 (72)	30 (100)	**<0.001**
LGE quantitative (%)	6.0 (0.0–9.0)	28.0 (16.0–44.0)	**<0.001**

ECV data were available for 62 of patients without and 31 with CA. LGE was available for 61 of patients without and 30 with CA. Bold values indicate significant differences.

4Ch, four-chamber view; IVSD, interventricular septum thickness end-diastolic; LAEDV, left atrial (LA) end-diastolic volume; LAESV, LA end-systolic volume; LVEDD, left ventricular (LV) end-diastolic diameter; LVEDV, LV end-diastolic volume; LVEF, LV ejection fraction; LVESD, LV end-systolic diameter; LVESV, LV end-systolic volume; LVMVI, LV mass-to-volume index; LVPWD, LV posterior wall thickness end-diastolic; LVSV, LV stroke volume.

Contrast application for ECV measurement was not performed in 19 patients for clinical reasons. Baseline characteristics and CMR measurements of LV structure and function were similar, but cardiac biomarkers were higher and eGFR lower in patients with ECV vs. those without ECV data (see Supplementary data online, *Table S2*). The data indicate advanced renal disease in patients without ECV rather than advanced ATTR-CM.

### Diagnostic performance of CMR parameters

ROC curve analysis (*Figure 2B*) demonstrated the highest diagnostic performance of native T1 obtained in the septal 4Ch orientation with an AUC of 0.836 (95% CI 0.760–0.913), followed by native T1 in the septal SAX (AUC 0.772, 95% CI 0.682–0.862), and the global T1 (AUC 0.713, 95% CI 0.613–0.812) value. The AUC of ECV (*n* = 93) was 0.904 (95% CI 0.825–0.982). Further AUC results of CMR parameters were 0.899 for LGE (95% CI 0.834–0.963), 0.768 for LV mass index (95% CI 0.676–0.861), and 0.776 for LVMVI (95% CI 0.688–0.864). Statistical comparisons between ROC curves demonstrated that ECV was superior compared with native T1 global and native T1 septal but similar to native T1 4Ch (*Table 3*).

**Table 3:** Comparison of AUC values between CMR parameter for the diagnosis of cardiac amyloidosis

Parameter 1	Parameter 2	*N*	AUC 1	AUC 2	*P*-value	Adjusted *P*-value
**T1 4Ch**	T1 septal	105	0.836	0.791	0.2154	0.377
**T1 4Ch**	T1 global	105	0.836	0.725	**0**.**0164**	0.0594
**T1 4Ch**	ECV	91	0.841	0.907	0.1316	0.2512
**T1 4Ch**	LVMI	103	0.834	0.771	0.3046	0.4569
**T1 4Ch**	LVMVI	103	0.834	0.773	0.3612	0.5057
**T1 4Ch**	LGE	89	0.844	0.897	0.249	0.4022
**T1 septal**	T1 global	112	0.772	0.712	**0**.**0177**	0.0594
**T1 septal**	ECV	93	0.791	0.907	**0**.**0036**	**0**.**0252**
**T1 septal**	LVMI	108	0.777	0.768	0.8926	0.9499
**T1 septal**	LVMVI	108	0.777	0.776	0.9954	0.9954
**T1 septal**	LGE	91	0.792	0.899	**0**.**0198**	0.0594
**T1 global**	ECV	93	0.706	0.907	**<0**.**0001**	**0**.**0001**
**T1 global**	LVMI	108	0.715	0.768	0.4428	0.547
**T1 global**	LVMVI	108	0.715	0.776	0.3935	0.5165
**T1 global**	LGE	91	0.704	0.899	**0**.**0002**	**0**.**0021**
**ECV**	LVMI	93	0.907	0.761	**0**.**0164**	0.0594
**ECV**	LVMVI	93	0.907	0.794	0.0661	0.1388
**ECV**	LGE	91	0.903	0.899	0.9047	0.9499
**LVMI**	LVMVI	108	0.768	0.776	0.8725	0.9499
**LVMI**	LGE	91	0.789	0.899	**0**.**0466**	0.1106
**LVMVI**	LGE	91	0.79	0.899	**0**.**0474**	0.1106

Bold values indicate significant differences. 4Ch, four-chamber; AUC, area under the curve; ECV, extracellular volume fraction; LGE late gadolinium enhancement; LVMI, left ventricular mass index; LVMVI, left ventricular mass-to-volume index.

On ROC curves, native T1 (4Ch) showed a sensitivity of 94.6% and a specificity of 69.1%, with the Youden’s index at 1346 ms. For ECV, the optimal cut-off ≥ 37% (Youden’s index) identified CA with a sensitivity of 83.9% and a specificity of 91.9%. Detailed diagnostic metrics are provided in Supplementary data online, *Table S3*, including PPV and NPV for an external pre-test probability of 13%, according to the literature.^4^ In a sensitivity analysis for ECV using multiple imputation, the AUC was 0.912 (±0.055), sensitivity 0.854, and specificity 0.930. In the best-case scenario, sensitivity was 0.839 and specificity 0.935. In the worst-case scenario, the corresponding values were 0.684 and 0.784, respectively.

In a multivariate logistic regression analysis, higher values of ECV [OR 1.352 (95% CI 1.202–1.585), *P* < 0.001], T1 4Ch [OR 1.015 (1.008–1.022), *P* < 0.001], T1 septal [OR 1.011 (1.005–1.018), *P* < 0.001], and T1 global [OR 1.010 (1.004–1.016), *P* = 0.002] were independently associated with the diagnosis of CA.

### Diagnostic algorithm by combining native T1 and ECV for the diagnosis of CA

**Figure 3:**
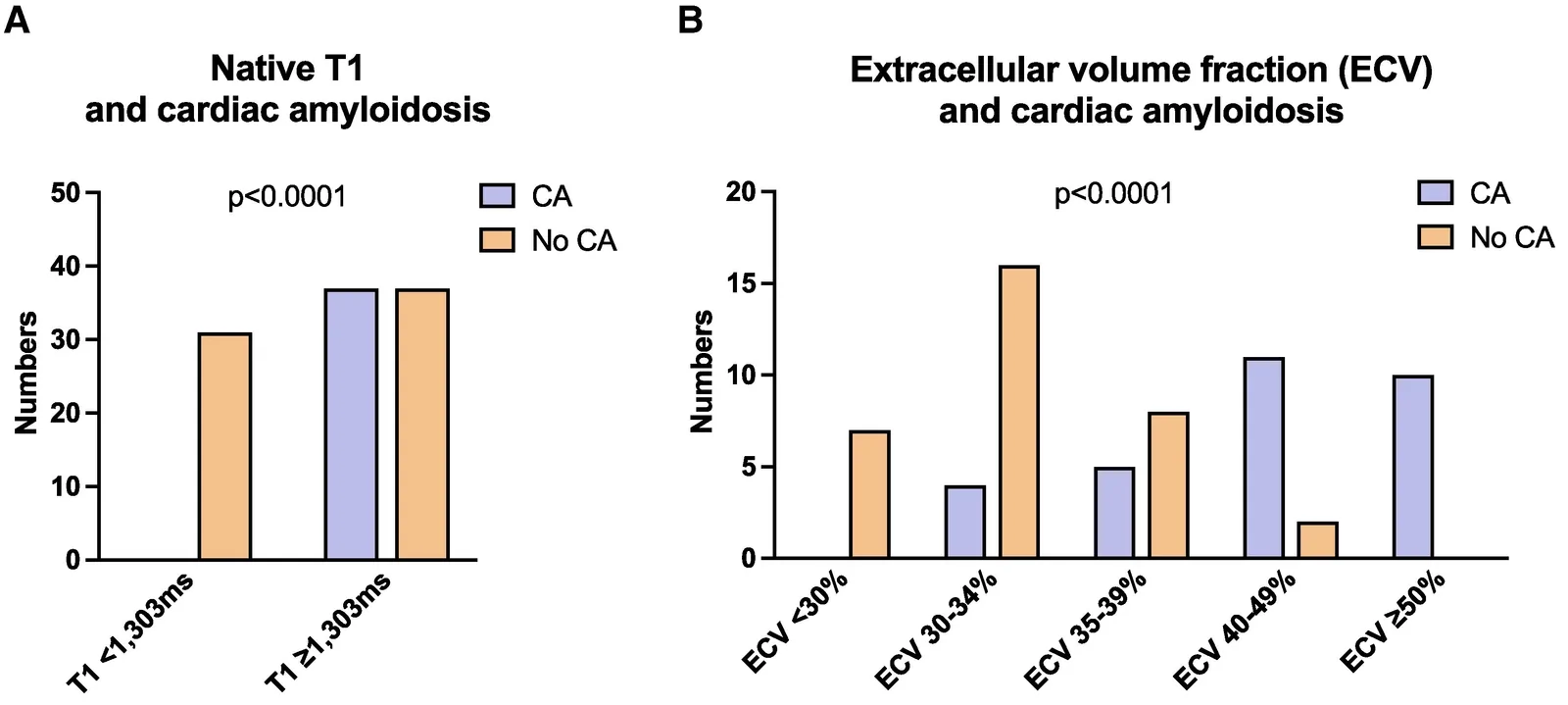
Native T1, ECV, and diagnosis of cardiac amyloidosis. Categories of (*A*) native T1 and (*B*) ECV and numbers of patients with (purple) and without (orange) CA. (*B*) includes patients with native T1 (4Ch) ≥ 1303 ms only. The analysis of the total study population without native T1 preselection revealed similar results (see Supplementary data online, *Figure S1*).

Sensitivity and specificity of any single CMR parameter were suboptimal. Native T1 < 1303 ms (*z*-score 3.0) excluded CA with 100% NPV (*Figure 3A*) while specificity was 45.6%. However, in patients with native T1 ≥ 1303 ms, the probability of CA increased with increasing ECV values (*P* < 0.0001; *Figure 3B*). Patients with ECV < 30% did not have CA as the underlying cause of HF (100% NPV). There was one female patient with ECV of 24% and Perugini 2 on DPD scan, but low ATTR amyloid burden on histology (5% myocardial area). Therefore, we considered CA as an incidental finding by study procedures, but not the reason for HF. On the contrary, ECV ≥ 50% confirmed CA (100% PPV). In patients with ECV 30–39%, both CA (occurring in 25%) and non-CA were frequent diagnoses, while the probability of CA significantly increased up to 85% when ECV was between 40 and 49%. Results were similar in the whole population, i.e. without selecting patients with T1 ≥ 1303 ms (see Supplementary data online, *Figure S1*).

**Figure 4:**
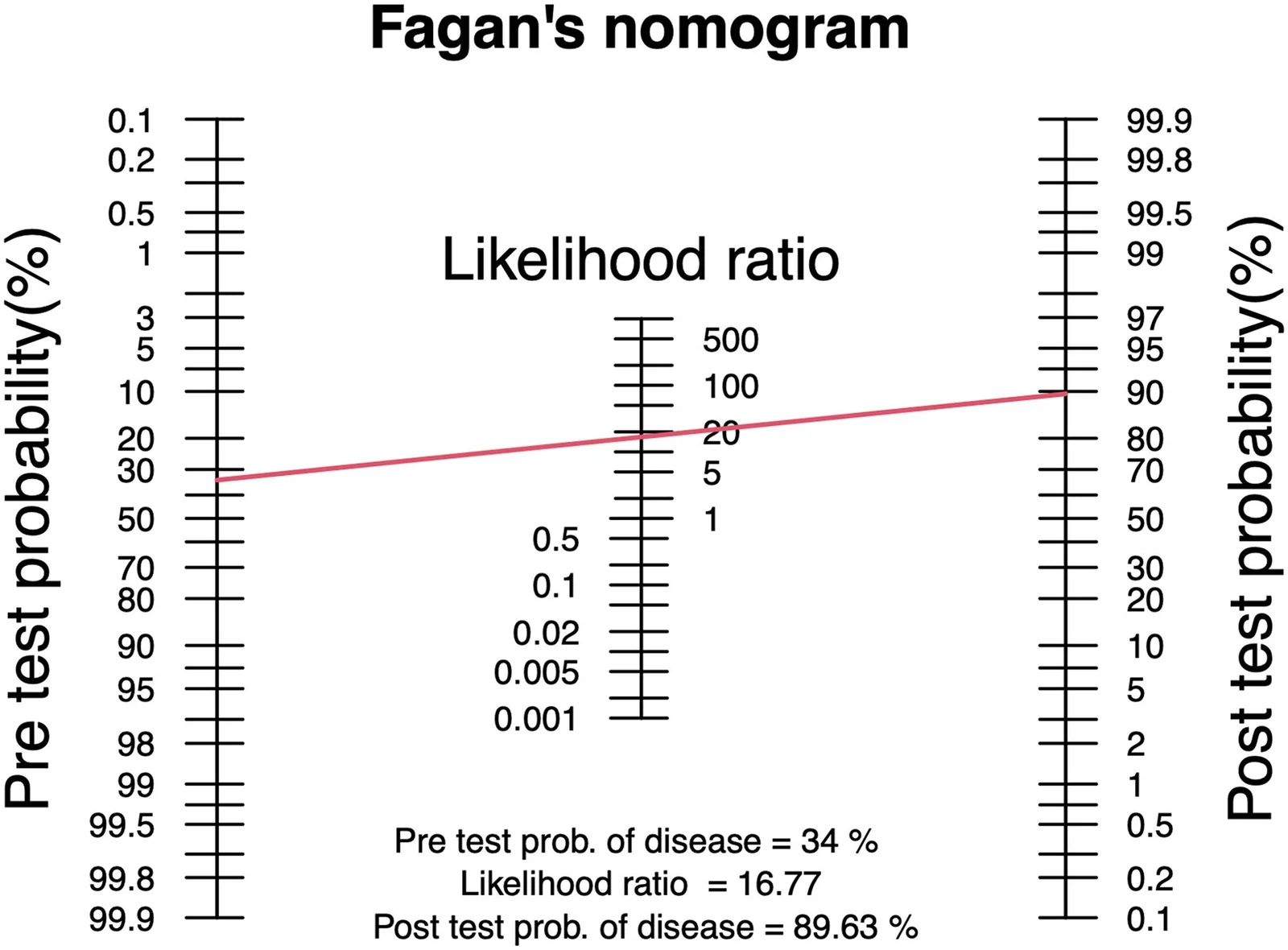
Fagan nomogram to facilitate clinical application of the study results in patients with different pre-test probabilities. The nomogram is based on combined T1 4Ch and ECV approach for the diagnosis of CA with an AUC of 0.907 (95% CI 0.8291–0.9848). The best threshold was identified at 0.44, providing a sensitivity of 84% and a specificity of 95%. In our population, 34% of patients had cardiac amyloidosis, i.e. pre-test probability of the disease was 34% (left). The likelihood ratio (middle) indicates that by using the diagnostic algorithm, a positive test is 17-fold more likely in CA compared with non-CA patients. This approach results in a post-test probability (right) of about 90%, i.e. the probability of CA is about 90% if both native T1 4Ch and ECV indicate CA.

The bootstrap-corrected AUC for the combined native T1 and ECV approach in the algorithm was 0.907 (95% CI 0.820–0.975). The combined native T1 and ECV approach yielded a net reclassification improvement for CA of 11.4% and 5.8%, respectively, compared with isolate T1 and ECV measurement. The Fagan nomogram (*Figure 4*) facilitates the translation of the results into populations with different pre-test probability of CA.

In the absence of external validation of the diagnostic algorithm, we performed leave-one-out (ROC 0.895, sensitivity 95%, specificity 74%) and *k*-fold cross-validation (AUC 0.867, sensitivity 95%, specificity 77%; *k* = 10) tests. They provided a more accurate estimate of performance and confirmed validation of the algorithm.

**Figure 5:**
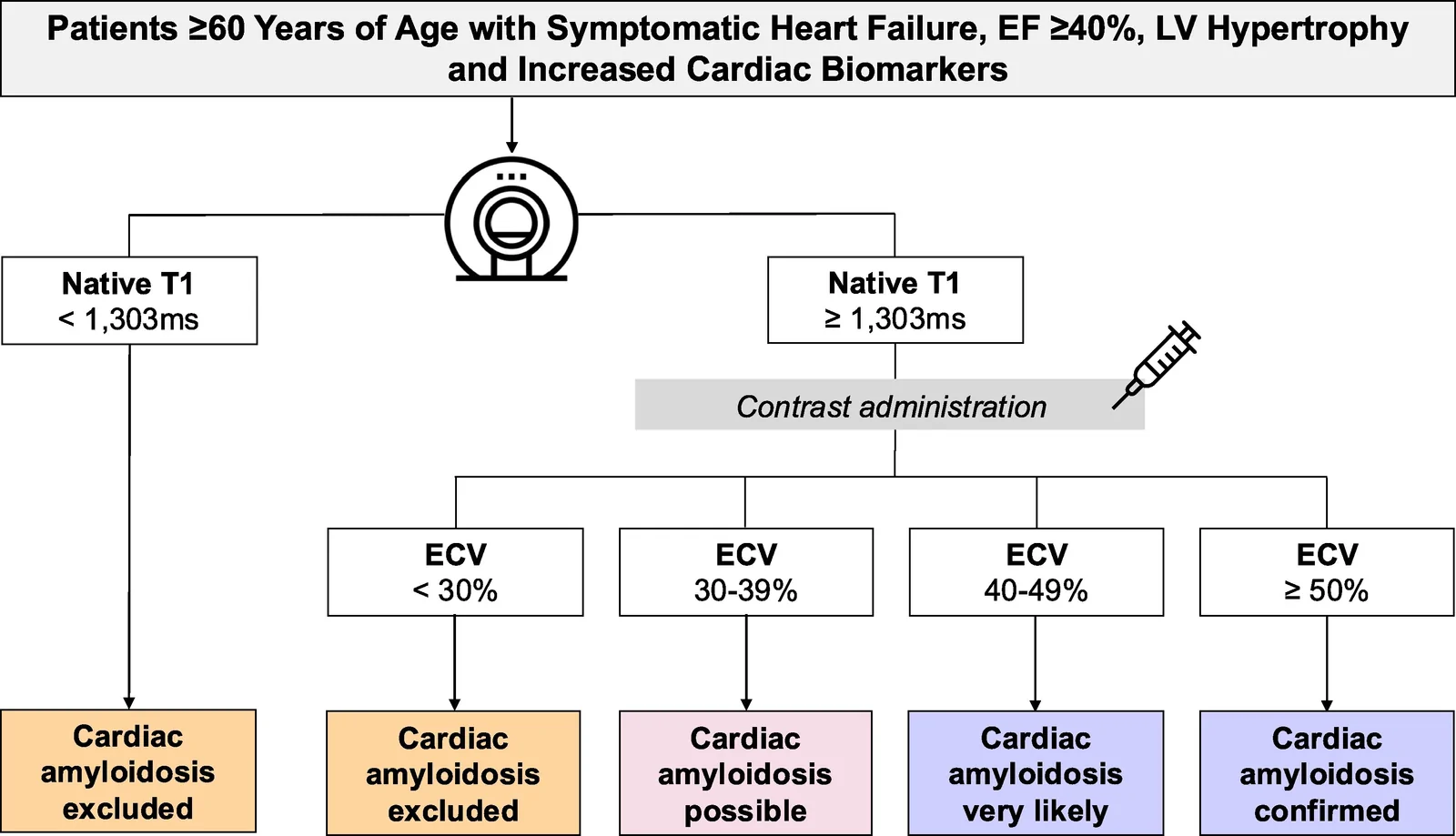
Diagnostic pathway. Cardiac amyloidosis can be excluded if native T1 is <1303 ms. The calculation of ECV requires contrast administration. ECV < 30% excluded cardiac amyloidosis as cause of HF, but incidental findings of early CA may occur. The higher ECV value, the more likely is the presence of CA, with ECV ≥ 50% confirming CA. This algorithm applies to patients included in this study, i.e. symptomatic HF, age ≥ 60 years, LVEF ≥ 40%, LV hypertrophy, and elevated cardiac biomarkers.

By this diagnostic sequence with native T1 and ECV, contrast administration could be avoided in 33%, i.e. those with native T1 < 1303 ms. A definite diagnosis of either confirmation or exclusion of CA could be provided in 50% of patients, i.e. those with native T1 < 1303 ms, native T1 ≥ 1303 ms and ECV <30%, and T1 ≥ 1303 ms and ECV ≥ 50%. We summarized the results of our study in *Figure 5* providing a diagnostic pathway for patients with HF, LVEF ≥ 40%, increased LV wall thickness, and elevated cardiac biomarkers.

## Discussion

This study investigated CMR with T1 imaging for the evaluation of CA in elderly patients with symptomatic HF and clinical risk factors for CA. The study provides three main findings: (i) there is a distinct CMR phenotype of amyloid cardiomyopathy with LV concentric remodelling with elevated native T1 values and higher ECV. (ii) For diagnostic purposes, native T1 < 1303 ms excluded CA. In patients with native T1 ≥ 1303 ms, the probability of CA increased with higher ECV values: ECV < 30% excluded CA, while all patients with ECV ≥ 50% had apparent CA. By this approach, CA was excluded or confirmed in about 50% of the patients. (iii) Since CA is underdiagnosed, systematic CMR may facilitate the screening for CA as the underlying cause of HF and to quantify cardiac remodelling in ATTR-CM.

Despite recent data reporting a CA prevalence of at least 16% among patients with HFpEF,^4^ CA is likely to remain underdiagnosed.^22^ The current cardiomyopathy guidelines from the European Society of Cardiology recommend to screen for CA.^23^ However, the systematic implementation of imaging in the diagnostic pathway of CA still needs to be determined. CMR enables the differentiation of hypertrophic phenotypes^13,19^ in subjects with elevated troponin levels.^24^ Previous studies investigating the role of CMR for the diagnosis of CA were performed in significantly pre-selected patients with high suspicion of CA, referred to a specialized amyloidosis centre.^16–18^ Thus, the role of CMR in unselected, i.e. without pre-existing suspicion of CA, elderly patients with stage C and D HF is unknown. Therefore, we prospectively studied the role of CMR with T1 mapping to diagnose CA in elderly patients with symptomatic HF NYHA class II to IV, increased LV wall thickness, and elevated cardiac biomarkers, i.e. patients at high risk for CA.

The ROC analyses showed that no single CMR parameter alone provided sufficient diagnostic accuracy. We observed best predictive values for native T1 measurement in the interventricular septum 4Ch orientation. An explanation may be that the septum is frequently affected by amyloid infiltration^23,25^ and less prone to artefacts compared with the lateral LV segments. This 4Ch T1 approach is supported by other CMR studies in CA^18,26,27^ and may evolve as standard for T1 mapping in CA. Superior diagnostic performance of ECV compared with native T1 has been reported.^15^ ECV is a measure of the myocardial interstitium, whereas T1 mapping is less specific and combines extra- and intracellular signals.^28^ This may explain better diagnostic performance of ECV. In our study, the optimal threshold of ECV for diagnosing CA was 37%. This ECV cut-off also has been identified in a recent study^18^ and in a meta-analysis.^15^ However, since cardiac amyloid deposition increases continuously, there are patients with CA and ECV ≤ 37%. Thus, a strict ECV cut-off would miss the diagnosis in patients with early amyloid disease.

To improve diagnostic utility of CMR, we combined native T1 and ECV into a diagnostic pathway for CA in the study population. Both native T1 and ECV are associated with the diagnosis of CA and statistically superior compared with one single CMR parameter. The high NPV (100%) of native T1 allowed to exclude CA in patients below the threshold of 1303 ms. In patients with native T1 ≥ 1303 ms, increasing ECV showed a consistent relationship with the probability of CA. In amyloid cardiomyopathy, ECV reflects myocardial amyloid burden and thus severity of the disease.^26^ An ECV ≥ 50% confirmed CA, and an ECV > 40% was strongly suspicious for CA. An ECV value between 30 and 39% is a grey zone without a concise diagnosis by CMR. Thus, these patients may have CA or fibrotic interstitial remodelling, for example, due to hypertensive heart disease. They should undergo bone scintigraphy to confirm or to exclude CA. Whether additional parameters such as LGE may improve the diagnostic yield of CMR in patients with ECV 30–39% should be analysed further. These data complement a recent larger but retrospective analysis where ECV ≥ 40% confirmed cardiac involvement in ATTR-CM.^26^

Our study provides new evidence for the diagnostic value of quantitative CMR in patients with symptomatic HF without previous suspicion of CA. A definite diagnosis by CMR, either confirmation or exclusion of CA, could be established in patients with distinct phenotypes. More importantly, CMR enabled screening for CA in patients at risk for CA, reflected by the high prevalence of CA in this study. The study underscores the importance of a systematic evaluation of underlying causes of HF to guide specific treatment of cardiomyopathies.

Our study included patients with HF and LVEF ≥ 40%. Thus, it complements a recent study from Güder *et al*. showing that CMR is a suitable option for diagnostic evaluation of patients with HF and LVEF < 40% to identify ischaemic aetiology.^29^ In that study, 50% of invasive coronary angiographies could have been avoided. Taken together, these data demonstrate that CMR provides useful imaging modality to evaluate the aetiology of HF non-invasively across the full spectrum of ejection fraction. Although the diagnostic discrimination of CMR is not perfect, i.e. uncertainty and need for further diagnostic workup may remain in some patients, it should be regarded as an important non-invasive part of multimodality cardiac imaging in HF to apply precision therapy. Our study demonstrates the impact of CMR to characterize ATTR-CM as recommended in the current guidelines.^30^ We provide evidence that unexplained HF in elderly patients with increased LV wall thickness and increased cardiac biomarkers deserves diagnostic evaluation whether amyloidosis may be the underlying cardiomyopathy.

### Limitations and strengths

The high prevalence of CA in our study (34%) may be explainable due to the requirement of elevated hs-cTnT and NT-proBNP levels at baseline. Furthermore, we included patients who fulfilled the inclusion criteria, but HF was not the primary reason for admission. One of the main strengths of our study (in contrast to most other CMR studies) is the consecutive inclusion of patients with symptomatic HF. Thus, the findings of our study primarily apply to patients in stage C and D heart failure. This is important since the particular cut-off values may be different in earlier stages of ATTR-CM. Mapping sequences do not cover the entire LV myocardium; hence, early stages of CA with regional amyloid accumulation may be missed by this approach. Contrast was applied to 95% of patients only for clinical reasons. Since only five patients suffered from AL amyloidosis in our study, the results are mainly applicable to patients with ATTR-CM and cannot be generalized to patients with AL. In the absence of validation in an independent external patient population, cross-validation techniques confirmed robustness of the diagnostic algorithm.

## Conclusion

The study showed that CMR-based evaluation with native T1 and ECV imaging provides important information to improve the diagnosis of CA. The CMR approach is validated and applicable to patients ≥ 60 years of age, symptomatic HF with LVEF ≥ 40%, increase LV wall thickness, and elevated cardiac biomarkers. These data support a broader use of CMR for evaluating patients with HF to identify underlying amyloidosis early and systematically.

## Supplementary Material

qyag131_Supplementary_Data

## Data Availability

The datasets analysed during the current study are available from the acorresponding author on reasonable request.

## Lead author biography

Daniel Lavall is a consultant, heart failure specialist and interventional cardiologist at Leipzig University Hospital, Germany. He is head of the local cardiac amyloidosis programme and the principal investigator of this and other clinical studies.
