# Supplementary material for: Cardiac magnetic resonance T1 mapping for amyloid cardiomyopathy in patients with heart failure
Source: Eur Heart J Imaging Methods Pract. 2026 Jul 23;4(3):qyag131. doi: 10.1093/ehjimp/qyag131 (PMC13521332; doi:10.1093/ehjimp/qyag131)
Supplement: Supplementary file 1 [file qyag131_supplementary_data.pdf]

Cardiac Magnetic Resonance Imaging for Screening of Amyloid Cardiomyopathy in Heart Failure

Supplement Material

Supplemental methods	Page 2
Supplement Table S1	Page 4
Supplement Table S2	Page 5
Supplement Table S3	Page 8
Supplement Figure S1	Page 10
References	Page 11

Methods

CMR protocol details

The sequence protocol was prefaced of survey images in coronal, sagittal, and transversal orientation. Retrospectively gated steady state free precession cine images were acquired in long and short axis orientation of the LV (repetition time (TR), 45ms; echo time (TE), 1.4ms; flip angle (FA), 45°; voxel size, 1.7 × 1.7 x 6.0mm^3). Native and post-contrast T1 values in basal, mid, and apical short axis slices as well as in one 4-chamber slice of the LV were measured using the modified look-locker inversion recovery sequence (TR, 2.1ms; TE, 0.98ms; FA, 20°; voxel size, 2.0 × 2.0 × 10.0mm^3). LGE was assessed at least 10min after intravenous contrast application in a short axis stack using phase-sensitive inversion recovery sequence (PSIR) (typical image parameters: TR, 3.53ms; TE, 956ms; FoV, 300mm; matrix, 256mm; voxel size, 0.6 × 0.6 × 10mm^3).

Reference methods for CA diagnosis

Technetium-99m-labelled 3,3-diphosphono-1,2-propanodicarboxylic acid ([^{99m}Tc]DPD) bone scintigraphy with SPECT/CT was performed in the department of nuclear medicine. As the diagnostic reference levels changed in 2021, standard activities of approximately 550 MBq [^{99m}Tc]DPD were intravenously injected in the first patients. After June 2021 the radiotracer activity was administered with 8 MBq [^{99m}Tc]DPD per kilogram bodyweight. Whole body images and single photon emission computed tomography/ computed tomography (CT) of the thorax were acquired 3 hours post injection at a gamma camera (Symbia Bold or Intevo, Siemens). The CT was performed in low-dose technique (auxiliary CT). The cardiac tracer retention was graded by an experienced physician blinded to the CMR results according to the visual Perugini scale from 0 to 3 points[22].

The screening for monoclonal protein included serum protein electrophoresis, serum immunofixation, and measurement of serum free light chains including kappa/lambda ratio.

Endomyocardial biopsy

Endomyocardial biopsy was performed through femoral access using a 6 French introducer sheet. 5 specimens from LV myocardium were obtained under fluoroscopic guidance by using a cardiac biopsy forceps. After the procedure, mechanical complications were excluded by echocardiography. The histological and immunohistological analyses of biopsies were performed externally.

Sample size calculation

A previous, retrospective study with 3T CMR revealed a sensitivity of 100% and a specificity of 97% of native T1 for the diagnosis of cardiac amyloidosis in patients with increased left ventricular wall thickness, when a cutoff of 1,341ms was used(1). Hence, diagnostic accuracy of native T1 was estimated 97.7%. The prevalence of cardiac amyloidosis among patients with symptomatic heart failure and increased LV wall thickness is about 13%(2). For sample size calculation, we assumed a significance level alpha = 5%, accuracy 97.5% and non-inferiority margin 5%. With the Power Analysis and Sample Size System software (PASS 2008, NCSS, LLC. Kaysville, Utah), we calculated a sample size of non-inferiority test for proportions n = 112 patients. In case of at least 108 correctly diagnosed patients (both, correct positive and correct negative for CA) by CMR, the primary endpoint would be met with a power of 85%.

Supplement Table S1: The RAISE score is a scoring system for the discrimination of patients with lone severe aortic valve stenosis and dual pathology of severe aortic valve stenosis and cardiac amyloidosis[1]. We included patients with a RAISE score of ≥ 2 points due to the high sensitivity for concomitant cardiac amyloidosis. Hs-cTnT, high-sensitivity cardiac troponin T.

Parameter	Points
**Carpal tunnel syndrome**	3
**Right bundle branch block**	2
**Age ≥ 85 years**	1
**hs-cTnT ≥ 20 ng/L**	1
**Interventricular septum ≥ 18 mm**	1
**If in sinus rhythm: E/A ratio > 1.4**	1
**If no bundle branch block or pacemaker: Sokolow index < 1.9 mV**	1

Supplement Table S2: Comparison of clinical and imaging characteristics between patients with ECV to those without ECV measurement

	**ECV measured (*n* = 93)**	**No ECV data (*n* = 19)**	***P* value**
**Age (years)**	82.0 (76.0-85.0)	76.0 (69.0-83.0)	0.131
**Female gender, *n* (%)**	42 (45)	8 (42)	1.000
**NYHA class**			0.083
**II, *n* (%)**	38 (41)	4 (21)	
**III, *n* (%)**	48 (63)	15 (79)	
**IV, *n* (%)**	0 (0)	7 (37)	
**Vital parameters**			
**Systolic blood pressure**	143±26	136±25	0.292
**(mmHg)**	78±18	77±13	0.797
**Diastolic blood pressure**	75 (66-85)	74 (61-78)	0.193
**(mmHg)**	25.3	27.8	0.874
**Heart rate (bpm)**	(23.7-29.7)	(22.8-29.6)	
**Body mass index (kg/m^2)**			
**Biomarkers**			
**NT-proBNP (ng/L)**	3,195 (2,333-5,845)	5,064 (3,350-9,351)	**0.042**
**hs-cTnT (ng/L)**	46.2 (31.2-71.8)	73.8 (45.1-129.5)	**0.035**
**eGFR ($mL/min/1.73m^2$)**	51.1±21.7	37.3±23.1	**0.014**
**Electrocardiogram**			
**Sinus rhythm, *n* (%)**	42 (45)	8 (42)	0.801
**Atrial fib./flutter, *n* (%)**	50 (54)	11 (58)	0.801
**AV block, *n* (%)**	16 (17)	2 (11)	0.538
**BBB, *n* (%)**	28 (30)	5 (26)	0.792
**Low voltage, *n* (%)**	11 (12)	4 (21)	0.494
**Echocardiography**			
**LV wall thickness (mm)**	14.0 (13.0-15.5)	14.5 (13.0-15.0)	0.416
**Pericardial effusion, *n* (%)**	17 (19)	3 (16)	1.000
**Comorbidities**			

**Hypertension, *n* (%)**	85 (91)	15 (79)	0.120
**Diabetes mellitus, *n* (%)**	39 (42)	10 (53)	0.452
**Atrial fib./flutter, *n* (%)**	65 (70)	15 (79)	0.580
**CAD, *n* (%)**	45 (48)	8 (42)	0.398
**TIA/stroke, *n* (%)**	14 (15)	3 (16)	1.000
**PAD, *n* (%)**	8 (9)	4 (21)	0.120
**CMR**			
**IVSD (mm)**	13.3 (11.2-17.0)	13.3 (11.3-14.5)	0.332
**LVPWD (mm)**	8.3 (7.0-10.0)	9.0 (8.0-10.0)	0.418
**LV wall mass index (g/m^2)**	59.0 (45.0-74.0)	54.0 (50.0-67.0)	0.597
**LVEDD (mm)**	46.0 (42.0-50.2)	46.0 (44.4-48.3)	0.947
**LVESD (mm)**	29.0 (24.0-36.5)	29.0 (26.5-32.2)	0.978
**LVEDV index (ml/m^2)**	46.0 (39.0-60.0)	52.0 (44.5-64.0)	0.295
**LVESV index (ml/m^2)**	17.0 (13.0-28.0)	18.0 (14.5-26.5)	0.585
**LVSV index (ml/m^2)**	28.0 (23.0-33.0)	31.0 (26.0-35.5)	0.205
**LVMVI (g/ml)**	1.24 (1.03-1.65)	1.08 (0.90-1.31)	**0.047**
**LVEF (%)**	62.0 (51.0-70.0)	63.0 (55.0-69.0)	0.883
**LAEDV index (ml/m^2)**	42.0 (27.5-52.0)	36.0 (30.0-50.0)	0.552
**LAESV index (ml/m^2)**	52.0 (35.0-63.5)	47.5 (43.0-60.0)	0.698
**T1 global (ms)**	1,277±77	1,312±93	0.081
**T1 septal, short axis (ms)**	1,265±91	1,303±87	0.101
**T1 septal, 4Ch (ms)**	1,360±89	1,396±90	0.165
**LGE qualitative, n (%)**	74 (81)	-	-
**LGE quantitative (%)**	8 (2-22)	-	-

Denial of contrast application during CMR was at the discretion of the physician responsible for CMR. 4Ch, four-chamber view; AV block, atrioventricular block; BBB, bundle branch block; bpm, beats per minute; CAD, coronary artery disease; ECG, electrocardiogram; ECV, extracellular volume fraction; eGFR, estimated glomerular filtration rate; hs-cTnT, high-sensitivity cardiac troponin T; IVSD, inter-ventricular septum thickness end-diastolic; LAEDV, left atrial (LA) end-diastolic volume; LAESV, LA end-systolic volume; LV, left ventricular; LGE, late gadolinium enhancement; LVEDD, left ventricular (LV) end-diastolic diameter; LVEDV, LV end-diastolic volume; LVEF, LV ejection fraction; LVESD, LVESV end-systolic

diameter; LVEDV, LV end-diastolic volume; LVMVI, LV mass-to-volume index; LVPWD, LV posterior wall thickness end-diastolic; LVSV, LV stroke volume; NT-proBNP, N-terminal pro-B-type natriuretic peptide; NYHA, New York Heart Association; PAD, peripheral arterial disease; TIA, transient ischemic attack.

Supplement Table S3: Diagnostic metrics of CMR parameters with their cutoff values for the diagnosis of cardiac amyloidosis.

	**Sensitivity**	**Specificity**	**PPV**	**NPV**	**PPV[13%*]**	**NPV[13%*]**	**PLR**	**NLR**	**DOR**
T1 global SAX ≥ 1,341ms	36.8% (21.8%-54.0%)	86.5% (76.5%-93.3%)	58.3% (40.7%-74.0%)	72.7% (67.3%-77.6%)	28.9%	90.1%	2.7 (1.3-5.6)	0.73 (0.6-1.0)	3.73 (1.5-9.5)
T1 septal SAX ≥ 1,341ms	36.8% (21.8%-54.0%)	83.8% (73.4%-91.3%)	53.8% (37.5%-69.4%)	72.1% (66.5%-77.1%)	35.8%	89.9%	2.3 (1.2-4.4)	0,75 (0.6-1.0)	3.0 (1.2-7.4)
T1 septal 4Ch ≥ 1,346ms	94.6% (81.8%-99.3%)	69.1% (56.7%-79.8%)	62.5% (53.7%-70.6%)	95.9% (85.8%-98.9%)	31.4%	98.8%	3.1 (2.1-4.4)	0.08 (0.02-0.30)	39.2 (8.6-178.2)
T1 septal 4Ch ≥ 1,303ms	100% (90.5%-100%)	45.6% (33.5%-58.1%)	50.0% (44.6%-55.4%)	100% (88.8%-100%)	21.6%	100%	1.8 (1.5-2.3)	0.00	63.0 (3.7-1067.8)
ECV ≥ 37%	83.9% (66.3%-94.6%)	91.9% (82.2%-97.3%)	83.9% (68.7%-92.4%)	91.9% (83.6%-96.23%)	60.7%	97.4%	10.4 (4.4-24.4)	0.18 (0.08-0.39)	59.3 (15.8-222.7)
LV mass index ≥ 62 g/m²	76.3% (59.8%-88.6%)	72.9% (60.9%-82.8%)	60.4% (50.0%-70.0%)	85.0% (75.9%-91.1%)	29.6%	95.4%	2.8 (1.8-4.3)	0.3 (0.2-0.6)	8.6 (3.5-21.6)
LV MVI ≥ 1.26 g/ml	56.7% (42.3%-70.7%)	84.2% (72.1%-92.5%)	76.3% (62.8%-86.0%)	68.6% (61.0%-75.3%)	34.9%	92.9%	3.6 (1.9-6.9)	0.5 (0.4-0.7)	7.0 (2.9-17.3)

All metrics are presented with the 95% confidence interval. Undiagnosed patients were count as negative when focusing on true positives (sensitivity and PPV) and as positive when focusing on true negatives (specificity and NPV). *Calculated with 13% pretest probability based on the prevalence of CA in the literature. 4Ch, four-chamber view; DOR, diagnostic odds ratio; Extracellular volume fraction (ECV); PLR, positive likelihood ratio; NLR, negative likelihood ratio; LV, left ventricular; LV MVI, LV mass-to-volume index; NPV, negative predictive value; PPV, positive predictive value; SAX, short axis.

Supplement Figure S1

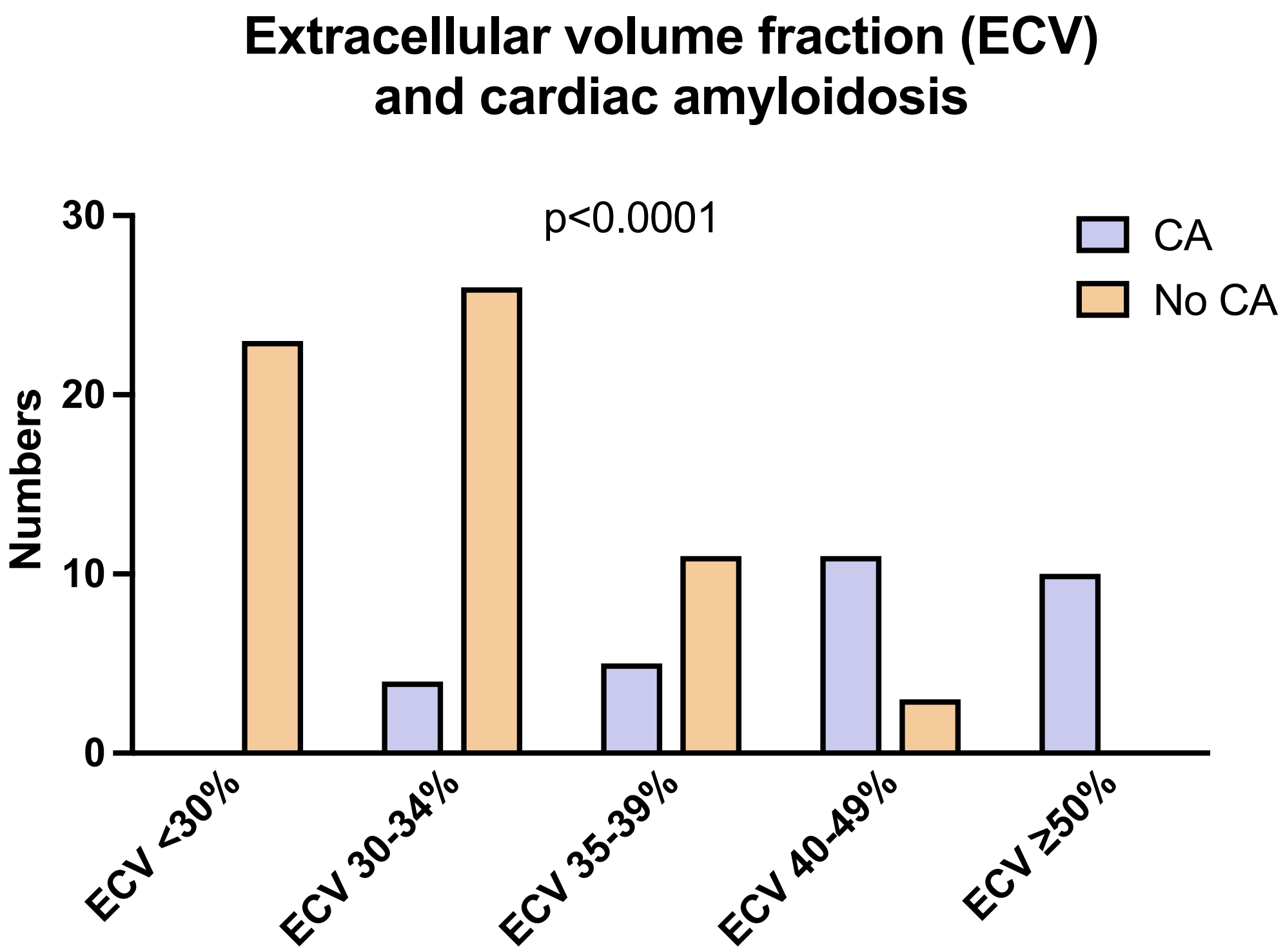

Figure S2: Extracellular volume fraction and diagnosis of cardiac amyloidosis in the study population

Numbers of patients with cardiac amyloidosis (CA; purple) and without (orange) according to categories of extracellular volume fraction (ECV)**.**

References

1. Lavall D, Vosshage NH, Geßner R, Stöbe S, Ebel S, Denecke T, et al. Native T1 mapping for the diagnosis of cardiac amyloidosis in patients with left ventricular hypertrophy. Clin Res Cardiol. 2023 Mar;112(3):334–42. doi:10.1007/s00392-022-02005-2

2. González-López E, Gallego-Delgado M, Guzzo-Merello G, De Haro-del Moral FJ, Cobo-Marcos M, Robles C, et al. Wild-type transthyretin amyloidosis as a cause of heart failure with preserved ejection fraction. Eur Heart J. 2015 Oct 7;36(38):2585–94. doi:10.1093/eurheartj/ehv338

3. Nitsche C, Scully PR, Patel KP, Kammerlander AA, Koschutnik M, Dona C, et al. Prevalence and Outcomes of Concomitant Aortic Stenosis and Cardiac Amyloidosis. Journal of the American College of Cardiology. 2021 Jan;77(2):128–39. doi:10.1016/j.jacc.2020.11.006

4. González-López E, Gallego-Delgado M, Guzzo-Merello G, De Haro-del Moral FJ, Cobo-Marcos M, Robles C, et al. Wild-type transthyretin amyloidosis as a cause of heart failure with preserved ejection fraction. Eur Heart J. 2015 Oct 7;36(38):2585–94. doi:10.1093/eurheartj/ehv338
